# FGF8 alleviates LPS-induced acute lung injury *via* the PPARγ/ERK1/2 signalling pathway in mice

**DOI:** 10.1080/07853890.2026.2706229

**Published:** 2026-08-03

**Authors:** Liangtao Song, Xuehui Liu, Qin Fang, Kai Yu, Danni Zhuo, Bin He, Changxing Chi, Chaoyou Lin, Mengyi Lin, Fanghua Gong, Yu Liu

**Affiliations:** a Department of Thoracic Surgery, The First Affiliated Hospital of Wenzhou Medical University, Wenzhou, China; b School of Pharmacy, Wenzhou Medical University, Wenzhou, China; c Department of Gynecology and Obstetrics, The Second Affiliated Hospital and Yuying Children’s Hospital of Wenzhou Medical University, Wenzhou, China

**Keywords:** FGF8, ALI, inflammation, apoptosis, fibrosis, PPARγ/ERK1/2 signalling pathway

## Abstract

**Background:**

Acute lung injury (ALI) and progressive acute respiratory distress syndrome (ARDS) have high mortality and limited effective therapies. Fibroblast growth factor 8 (FGF8) participates in lung development, inflammation and cell regulation, yet its function in ALI remains unclear.

**Methods:**

We constructed an LPS-induced mouse ALI model to detect FGF8 expression. FGF8 haploinsufficiency and overexpression systems were used to explore its biological function. RNA-seq combined with GO and KEGG enrichment analyses screened target pathways, which were further verified via experiments. The PPARγ antagonist GW9662 was applied to confirm the regulatory role of PPARγ.

**Results:**

Lung FGF8 expression was significantly upregulated in LPS-challenged mice. FGF8 haploinsufficiency exacerbated weight loss, pulmonary edema, lung histological injury, pro-inflammatory cytokine (IL-1β, IL-6, TNF-α) release, and apoptosis imbalance. It also accelerated collagen accumulation and elevated profibrotic markers including α-SMA, TGF-β1 and COL1A1. Conversely, FGF8 overexpression relieved all these pathological phenotypes. Mechanistically, FGF8 haploinsufficiency inhibited PPARγ and activated ERK1/2 phosphorylation, while FGF8 overexpression reversed this change. Blocking PPARγ with GW9662 further boosted ERK1/2 activation and abolished the protective effects of FGF8 against ALI.

**Conclusion:**

FGF8 alleviates inflammation, apoptosis and early pulmonary fibrosis in LPS-induced ALI mice via the PPARγ/ERK1/2 signaling pathway.

## 1. Introduction

Acute lung injury (ALI)/acute respiratory distress syndrome (ARDS) is an acute respiratory disorder resulting from sepsis induced by diverse intra- and extrapulmonary factors, including bacterial infection, endotoxins and trauma [1]. Its pathological features include inflammatory cell infiltration, increased vascular and epithelial permeability, interstitial oedema and damage to the alveolar septa [2]. Despite the application of mechanical ventilation, glucocorticoids and fluid management, the mortality rate of ARDS remains approximately 35%–46%, with no currently effective therapeutic strategies available [3]. Lipopolysaccharide (LPS) activates Toll-like receptor 4 (TLR4), triggering downstream signalling pathways and inducing the release of large quantities of proinflammatory cytokines [4]. Persistent inflammation and tissue injury promote alveolar epithelial cell apoptosis and dysregulated fibroblast-mediated repair, ultimately progressing to pulmonary fibrosis and impairing patient recovery and prognosis [5].

Fibroblast growth factor (FGF) is a family of over 20 polypeptide signalling molecules that modulate development, metabolism and tissue repair through paracrine and endocrine mechanisms [6]. FGF8, a representative paracrine factor, participates in lung development and is essential for alveolar formation [7]. During sepsis, elevated FGF8 expression has been reported to mitigate lung inflammation through immune regulation [8]. Moreover, under serum-free or hypoxic conditions, apoptosis is elevated, whereas FGF8 supplementation reduces apoptotic rates [9]. Although FGF8 is implicated in inflammatory regulation and cell survival, its function in ALI remains insufficiently defined.

This study demonstrated that FGF8 expression was elevated in an LPS-induced mice ALI model. FGF8 haploinsufficiency intensified lung inflammation, apoptosis and early pulmonary fibrotic response, whereas FGF8 overexpression attenuated these pathological changes. Further analysis indicated that FGF8 alleviated LPS-induced ALI by upregulating PPARγ and suppressing ERK1/2 activation.

## 2. Materials and methods

### 2.1. Experimental materials

LPS (Cat. # L8880) was purchased from Solarbio Science & Technology Co., Ltd. (Beijing, China). AAV9-FGF8 was purchased from Shanghai GeneChem Co., Ltd. (Shanghai, China). GW9662 (Cat. # HY-16578) was purchased from MedChemExpress (Shanghai, China). Mouse Direct PCR Kit (For Genotyping) was purchased from Selleck China (Shanghai, China). FreeZol Reagent (Cat. # R711-01/02), HiScript^®^IV All-in-One Ultra RT SuperMix (Cat. # R433-01) and Taq Pro Universal SYBR qPCR Master Mix (Cat. # Q712-02/03) were purchased from Vazyme (Nanjing, China). 180 kDa prestained protein parker(Cat. # MP102-02) was purchased from Vazyme (Nanjing, China). Thermo Scientific GeneRuler 100 bp DNA Ladder (Cat. # SM0241) was purchased from Thermo Fisher Scientific (Shanghai, China). Antibodies included: Anti-FGF8 (1:1000; Cat. # SC-293479) was purchased from Santa Cruz Biotechnology (TX, USA). Anti-Bcl-2 (1:1000; Cat. # 381702) was purchased from Zenbio Biotechnology Company (Chengdu, China). Anti-Cleaved caspase-9 (1:1000; Cat. # 9509) was purchased from Cell Signaling Technology (Boston, MA, USA). Anti-COL1A1 (1:1000; Cat. # Ab270993) was purchased from Abcam (Cambridge, UK). Anti-α-SMA (1:750; Cat. # AF1032) and Anti-TGF-β1 (1:750; Cat. # AF1027) were purchased from Affinity (CT, USA). Anti-ERK1/2 (1:1000; Cat. # RA2201) and Anti-Phospho-ERK1/2 (1:1000; Cat. # RA2202) were purchased from Vazyme Biotech Co., Ltd. (Nanjing, China). Anti-Bax (1:1000; Cat. # 60267-1-Ig), Anti-PPARa (1:1000; Cat. # 66826-1-Ig), Anti-PPARδ (1:1000; Cat. # 60193-1-Ig), Anti-PPARγ (1:1000; Cat. # 66936-1-Ig), Anti-GAPDH (1:5000; Cat. # 60004-1-Ig), HRP-conjugated Affinipure Goat Anti-Mouse IgG(H + L) (1:10000; Cat. # SA00001-1) and HRP-conjugated Affinipure Goat Anti-Rabbit IgG(H + L) (1:10000; Cat. #SA00001-2) were purchased from Proteintech Group, Inc. (Wuhan, China). All antibodies were stored at −20 °C prior to use.

### 2.2. Experimental Animals and ALI model establishment

#### 2.2.1. Animals and ethics approval

C57BL/6J male mice were obtained from Zhejiang Charles River Laboratory Animal Technology Co., Ltd., and FGF8^+/−^ male mice on a C57BL/6J background were sourced from laboratory-bred colonies (Wenzhou Medical University, Wenzhou, China). All animals were maintained under SPF conditions with unrestricted access to food and water. Each mice serves as an independent experimental unit, housed 5 per cage. Experimental procedures were conducted in strict compliance with the regulations for the care of laboratory animals and approved by the Animal Ethics Committee of Wenzhou Medical University (wydw-2025-0170).

#### 2.2.2. Mice grouping and model establishment

First, 6–8-week-old mice were randomly allocated into control (Ctrl), low-dose (LpsL) and high-dose (LpsH) groups. The Ctrl group received no intervention, while the LpsL and LpsH groups were administered intraperitoneal injections of LPS at 5 mg/kg and 10 mg/kg, respectively, to induce ALI [10,11]. Next, 6–8-week-old FGF8^+/+^ and FGF8^+/−^ mice were assigned to control (Ctrl), model (LPS) and FGF8 haploinsufficiency (FGF8^+/−^) groups. The Ctrl group received no intervention, while the LPS and FGF8^+/−^ groups were injected intraperitoneally with LPS (5 mg/kg) to establish the ALI model. Then, 3–4-week-old mice were randomized into control (Ctrl), model (LPS), negative vehicle (Vehicle) and overexpression (FGF8-OE) groups. The Vehicle and FGF8-OE groups were administered AAV9-Vehicle and AAV9-FGF8 (2 × 10^11^) *via* tail vein injection, respectively. After 3 weeks of stable gene expression, the Ctrl group remained untreated, whereas the remaining groups received intraperitoneal LPS (5 mg/kg) to induce ALI. Finally, 3–4-week-old mice were divided into vehicle (Vehicle), overexpression (FGF8-OE) and inhibitor (GW9662) groups. The Vehicle group received AAV9-Vehicle *via* tail vein injection, while the FGF8-OE and GW9662 groups received AAV9-FGF8. Following 3 weeks of stable expression, all mice were intraperitoneally injected with LPS (5 mg/kg) to induce ALI. Additionally, the GW9662 group received an intraperitoneal injection of GW9662 (1 mg/kg, dissolved in 10% DMSO) 1 h prior to LPS administration [12,13].

All groups (5–6 mice per group) were euthanized under isoflurane anaesthesia 24 h after the establishment of untreated or induced ALI models, and serum along with lung tissue samples were harvested for further analysis.

### 2.3. Genotyping of FGF8^+/−^ mice

Tail tip samples were collected 14 days postnatally. Genomic DNA was isolated using a Mouse Direct PCR Kit (Selleck, USA) following the manufacturer’s protocol and subsequently amplified using a PCR system (T100, Bio-Rad, Hercules, CA, USA). Agarose gel was prepared by dissolving 2 g of agarose in 100 ml of 1 × TAE buffer, heating to complete dissolution, cooling and adding a green fluorescent nucleic acid dye (Cat. # AC11937, Shanghai Jizhi Biochemical Technology Co., Ltd., Shanghai, China). A 10 μL volume of PCR product was electrophoresed on the agarose gel, and band patterns were detected using an automated imaging analysis system (IQ800, GE, USA). A single band indicated FGF8^+/+^ genotype, whereas the presence of two distinct bands indicated FGF8^+/−^ genotype. Primer sequences: FGF8^+/−^, forward: 5′-GAGCACGACATTCCACGAGC-3′, reverse: 5′-CGCGCCTGAAGATATAGAAGATA-3′.

### 2.4. Lung Wet-to-dry weight ratio

The left lung was excised, and surface blood and fluid were gently removed using filter paper. The wet weight was immediately measured. Lung tissue was then dehydrated in an oven (DGG-9070A, Shanghai Senxin Laboratory Instrument Co., Ltd., Shanghai, China) at 65 °C until a constant weight was achieved and subsequently recorded as the dry weight. The wet-to-dry weight ratio was calculated to quantitatively evaluate pulmonary oedema severity.

### 2.5. Histopathological analysis (H&E staining)

The right lower lung lobe was excised and fixed in 4% paraformaldehyde. Following fixation, samples underwent sequential dehydration with graded ethanol, xylene clearing, paraffin embedding and sectioning at a thickness of 5 μm (Paraffin embedding station and rotary microtome, histocorebiocut, Leica, Germany). After dewaxing and rehydration, sections were subjected to haematoxylin and eosin (H&E) staining (Solarbio, China) according to the manufacturer’s protocol. Stained sections were examined using an upright fluorescence microscope (Eclipse80i, Nikon, Japan), and images were captured. Lung tissue injury was semiquantitatively evaluated by a blinded observer using a 0–4 scale based on alveolar congestion or haemorrhage, neutrophil infiltration, alveolar wall thickening and hyaline membrane formation. Scoring parameters were defined as follows: 0, no injury; 1, <25% injury; 2, 25–50%; 3, 50–75%; and 4, >75%. Multiple randomly selected fields per section were analysed, and mean scores were calculated.

### 2.6. Masson trichrome staining

Paraffin-embedded lung sections (5 μm) were dewaxed, rehydrated and stained using the Masson trichrome kit (Solarbio, China) according to the provided instructions. Stained sections were examined under an upright fluorescence microscope (Eclipse80i, Nikon, Japan), and images were documented. Collagen fibre deposition (blue-stained areas) was analysed semiquantitatively using ImageJ software (National Institutes of Health, Hesda, MD, USA), and the proportion of collagen area relative to the total field was calculated.

### 2.7. ELISA

Serum samples were collected from mice, and IL-1β, IL-6 and TNF-α levels were determined using ELISA kits (Jiangsu Meimian Biotechnology Co., Ltd., China) following the respective protocols.

### 2.8. qRT-PCR

Total RNA was isolated from lung tissue using FreeZol Reagent (Vazyme, China) following the manufacturer’s protocol. RNA concentration and purity were verified using a NanoDropOne micro-spectrophotometer (NanoDropOne, Thermo, USA). Subsequently, 1 μg of total RNA was reverse-transcribed into cDNA using the HiScript^®^ IV All-in-One Ultra RT SuperMix. Quantitative PCR was conducted with Taq Pro Universal SYBR qPCR Master Mix on a fluorescent quantitative PCR system (CFX384, Bio-Rad, USA). GAPDH served as the internal control, and relative gene expression levels were calculated using the 2^-ΔΔCt^ method. Primer sequences: IL-1β, forward: 5′-GCAACTGTTCCTGAACTCAACT-3′, reverse: 5′-TCAACTGCCTGGGGTTTTCTA-3′; IL-6, forward: 5′-TAGTCCTTCCTACCCCAATTTCC-3′, reverse: 5′-CTTCCTCACCGATTCCTGGTT-3′; TNF-α, forward: 5′-CCCTCACACTCAGATCATCTTCT-3′, reverse: 5′-GACATCGGGTGCAGCATCG-3′; FGF8, forward: 5′-AGGGGAAGCTAATTGCCAAGA-3′, reverse:5′-TACCGGAAATGGGCGTTTCC-3′; GAPDH, forward: 5′-AGGTCGGTGTGAACGGATTTG-3′, reverse:5′-ACTGGAGTTGATGTACCAGATGT-3′.

### 2.9. Western blot

Lung tissue samples were lysed on ice in RIPA buffer supplemented with protease and phosphatase inhibitors, followed by centrifugation to obtain the supernatant. Protein concentration was measured using the BCA assay. Equal amounts of protein were mixed with loading buffer, heat-denatured, subjected to 10% SDS-PAGE (Electrophoresis apparatus, Bio-Rad, USA), and transferred onto PVDF membranes *via* wet transfer. Membranes were blocked with 5% skim milk or BSA in TBST for 2 h at room temperature and then incubated overnight at 4 °C with appropriately diluted primary antibodies. After three washes with TBST (7 min each), membranes were incubated with HRP-conjugated secondary antibodies for 1 h at room temperature, washed again three times and visualized using enhanced ECL reagents according to the manufacturer’s instructions. Images were captured using an automated imaging analysis system (IQ800, GE, USA), and the grayscale intensity of protein bands was quantified using ImageJ software.

### 2.10. RNA-seq

Total RNA was isolated from lung tissue using FreeZol Reagent (Vazyme, China) following the manufacturer’s protocol. RNA integrity was verified with the Bioanalyzer 2100 system and RNA6000 Nano LabChip Kit (5067-1511, Agilent, USA), ensuring a RIN value above 7.0. Qualified samples were used to construct sequencing libraries, followed by paired-end sequencing (2 × 150 bp) on the Illumina Novaseq^™^ 6000 platform (LC-Bio Technology CO., Ltd., Hangzhou, China). Post-quality control, raw sequencing reads were processed to identify differentially expressed genes (DEGs) using DESeq2 with thresholds of false discovery rate (FDR) < 0.05 and absolute fold change (|log_2_FC|) > 2. Subsequent functional annotation included Gene Ontology (GO) and Kyoto Encyclopedia of Genes and Genomes (KEGG) enrichment analyses of the Differentially Expressed Genes (DEGs).

### 2.11. Statistical analysis

Data are expressed as mean ± SEM and analysed using GraphPad Prism 8.0 (GraphPad Software, San Diego, CA, USA). Group comparisons were conducted using unpaired t-tests or one-way ANOVA. Each experiment was performed at least three times, and statistical significance was defined as *p <* 0.05.

## 3. Results

### 3.1. FGF8 expression was upregulated in the LPS-induced ALI mice model

**Figure 1.**
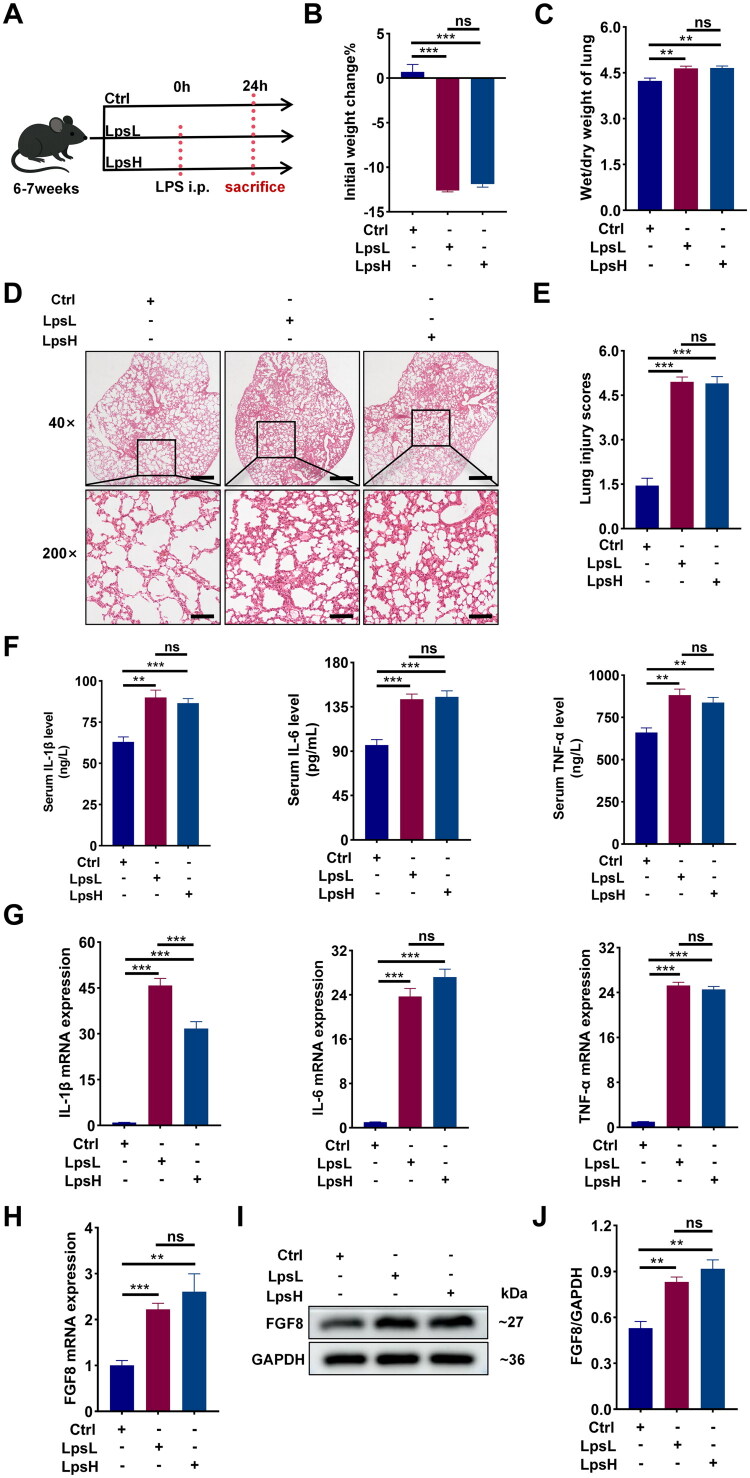
**FGF8 expression was upregulated in the LPS-induced ALI mice model**. **(A)** Animal experimental design diagram; **(B)** body weight changes at 24 h following untreated/LPS intervention (*n* = 5); **(C)** lung wet-to-dry weight ratio (*n* = 5); **(D)** haematoxylin–eosin staining of paraffin-embedded lung sections (40×, scale bar = 500 μm; 200×, scale bar = 100 μm); **(E)** quantitative assessment of lung histopathological injury (*n* = 5); **(F)** serum levels of IL-1β, IL-6 and TNF-α detected by ELISA (*n* = 4–5); **(G)** qRT-PCR analysis of lung tissue IL-1β, IL-6 and TNF-α mRNA levels (*n* = 4–5); **(H)** qRT-PCR analysis of FGF8 mRNA in lung tissue (*n* = 4–5); **(I, J)** Western blot analysis of FGF8 protein in lung tissue. Ctrl: control group; LpsL: low-dose LPS group; LpsH: high-dose LPS group. ns: not statistically significant, *p < 0.05, **p < 0.01, ***p < 0.001.

ALI was induced in mice through intraperitoneal administration of 5 mg/kg and 10 mg/kg LPS (Figure 1(A)). Both the LpsL and LpsH groups showed marked reductions in body weight (Figure 1(B), *p <* 0.001) and elevated lung wet-to-dry weight ratios (Figure 1(C), *p <* 0.01) relative to the Ctrl group, reflecting aggravated pulmonary oedema. H&E staining demonstrated preserved alveolar structure without inflammatory infiltration or intraalveolar haemorrhage in the Ctrl group, whereas the LpsL and LpsH groups displayed substantial alveolar collapse, thickening of the alveolar wall, inflammatory cell infiltration and intraalveolar haemorrhage (Figure 1D). Correspondingly, the overall pathological injury score, derived from alveolar structural damage, inflammatory infiltration, intraalveolar haemorrhage and hyaline membrane formation, was significantly elevated in both the LpsL and LpsH groups compared with the Ctrl group (Figure 1(E), *p <* 0.001). Serum concentrations of IL-1β, IL-6 and TNF-α were markedly increased in the LpsL and LpsH groups (Figure 1(F), *p <* 0.01), accompanied by elevated mRNA expression of these proinflammatory cytokines in lung tissue (Figure 1(G), *p <* 0.001), with IL-1β mRNA showing a particularly prominent rise in the LpsL group. In addition, FGF8 mRNA and protein levels were markedly increased in lung tissue in both the LpsL and LpsH groups (Figure 1(H)-(J), *p <* 0.01). Collectively, both 5 mg/kg and 10 mg/kg LPS effectively established an ALI mice model without notable phenotypic differences between the two doses. The 5 mg/kg dose was thus selected for subsequent experiments. The substantial induction of FGF8 in ALI indicates a potential role in the disease process, warranting further mechanistic investigation.

### 3.2. FGF8 haploinsufficiency exacerbated LPS-induced ALI

**Figure 2.**
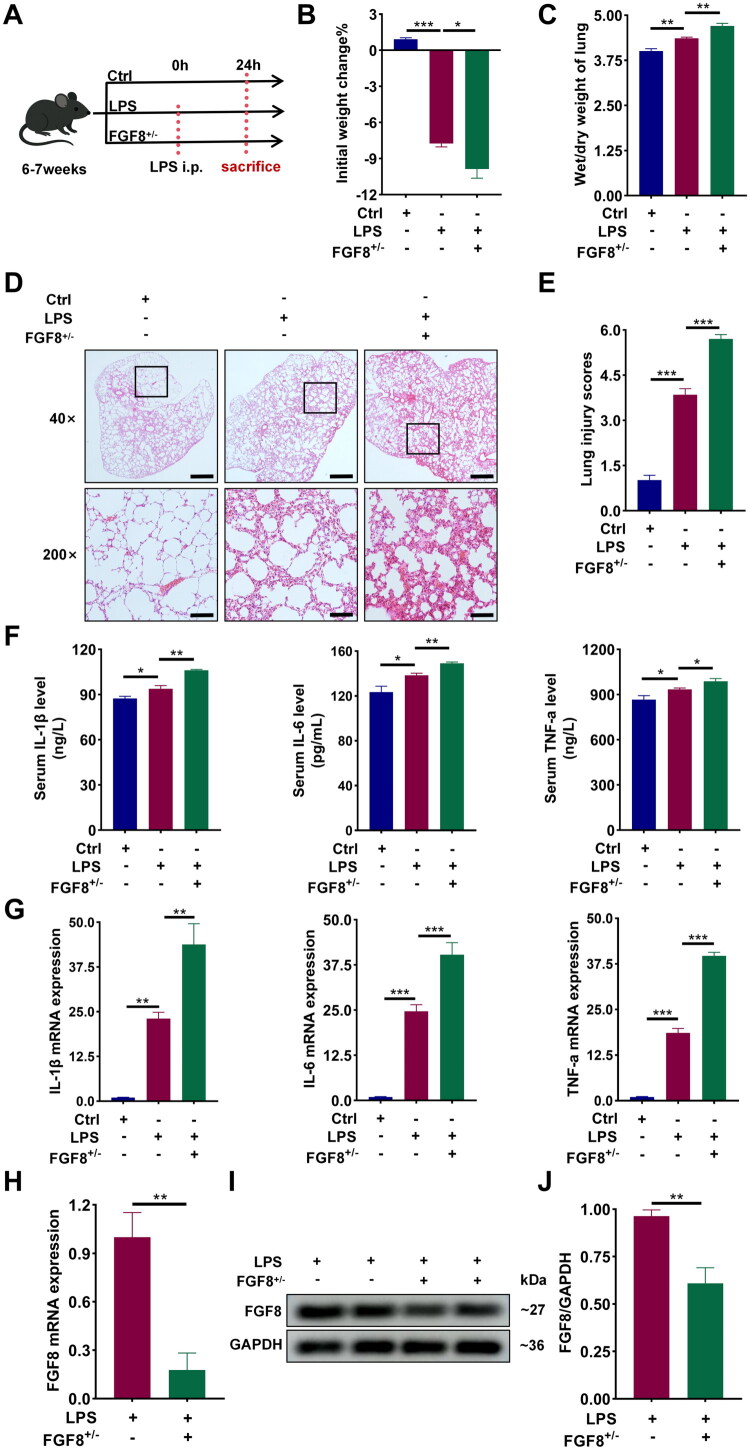
**FGF8 haploinsufficiency exacerbated LPS-induced ALI**. **(A)** Animal experimental design diagram; **(B)** body weight changes at 24 h following untreated/LPS intervention (*n* = 5); **(C)** lung wet-to-dry weight ratio (*n* = 5); **(D)** haematoxylin–eosin staining of paraffin-embedded lung sections (40×, scale bar = 500 μm; 200×, scale bar = 100 μm); **(E)** histopathological scoring (*n* = 5); **(F)** serum IL-1β, IL-6 and TNF-α levels measured by ELISA (*n* = 4–5); **(G)** qRT-PCR analysis of inflammatory cytokine mRNA in lung tissue (*n* = 4–5); **(H)** qRT-PCR evaluation of FGF8 mRNA in lung tissue (*n* = 5); **(I, J)** Western blot analysis of FGF8 protein expression. Ctrl: control group; LPS: model group; FGF8±: FGF8 haploinsufficiency group. *p < 0.05, **p < 0.01, ***p < 0.001.

Due to the lethality of FGF8 knockout (FGF8^-/-^) mice, the impact of partial FGF8 loss on ALI was investigated in heterozygous FGF8± mice (Figure 2(A), and genotype identification shown in Fig. S1A). Compared with the LPS group, FGF8± mice exhibited greater body weight loss (Figure 2(B), *p <* 0.05) and a further increase in lung wet-to-dry weight ratio (Figure 2(C), *p <* 0.01). H&E staining demonstrated more extensive alveolar collapse, thickened alveolar wall, intensified inflammatory cell infiltration and increased alveolar haemorrhage in the FGF8± group (Figure 2(D)). Correspondingly, pathological injury scores were significantly higher (Figure 2(E), *p <* 0.001). Serum levels of IL-1β, IL-6 and TNF-α were elevated in the FGF8± group (Figure 2(F), *p <* 0.05), accompanied by increased mRNA expression of these cytokines in lung tissue (Figure 2(G), *p <* 0.01). Western blot and qRT-PCR analyses confirmed that FGF8 mRNA and protein levels in lung tissue were markedly reduced in the FGF8± group compared with the LPS group (Figure 2(H)-(J), *p <* 0.01). Collectively, FGF8 haploinsufficiency significantly intensifies LPS-induced ALI.

### 3.3. FGF8 haploinsufficiency promoted lung cell apoptosis and pulmonary fibrosis

**Figure 3.**
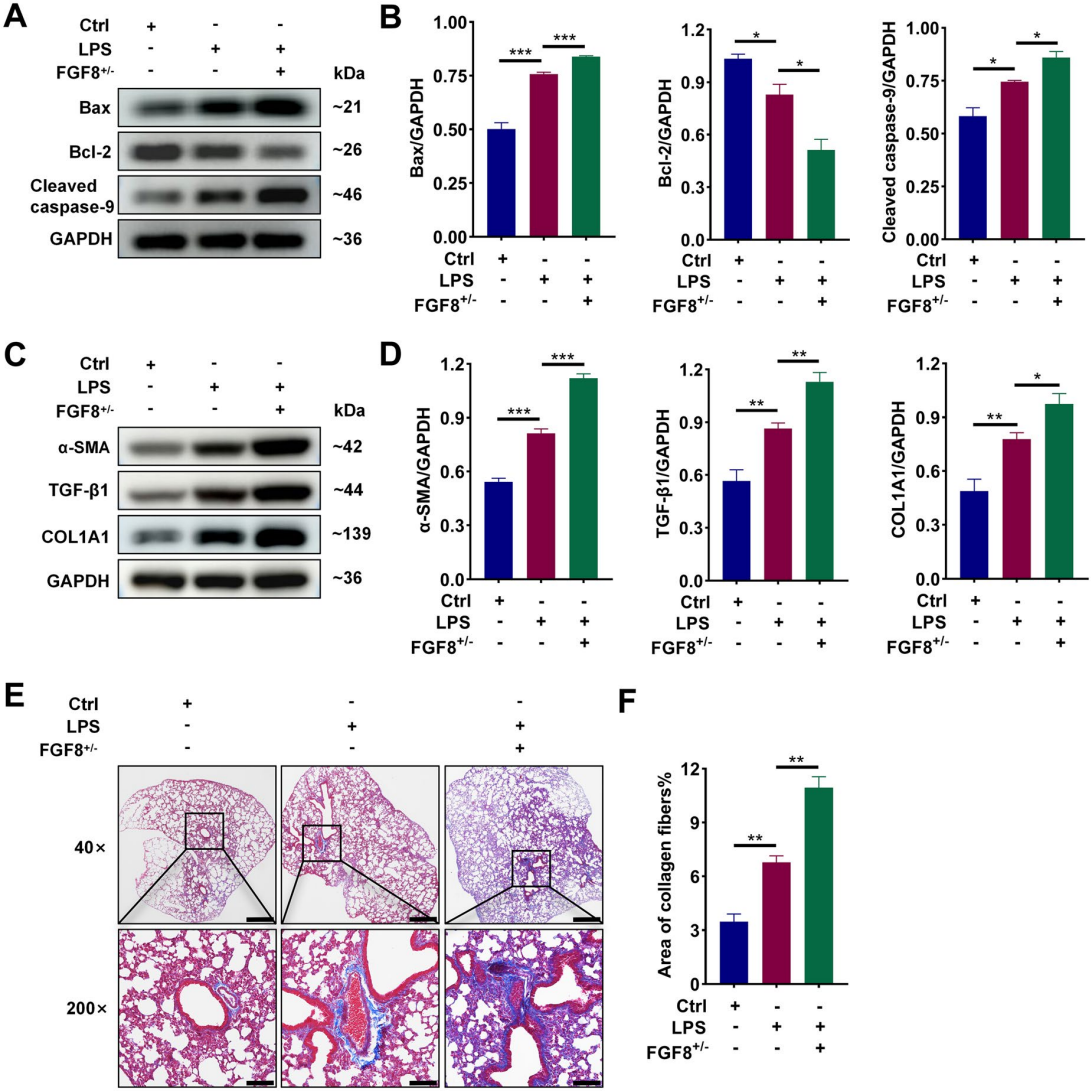
**FGF8 haploinsufficiency promoted lung cell apoptosis and pulmonary fibrosis**. **(A, B)** Western blot analysis of Bax, Bcl-2 and Cleaved caspase-9 in lung tissue; **(C, D)** Western blot analysis of α-SMA, TGF-β1 and COL1A1 protein expression; **(E)** Masson staining of collagen fibers in paraffin-embedded lung sections (40×, scale bar = 500 μm; 200×, scale bar = 100 μm); **(F)** semi-quantitative evaluation of collagen deposition. *p < 0.05, **p < 0.01, ***p < 0.001.

The impact of FGF8 haploinsufficiency on lung cell apoptosis and early pulmonary fibrotic response was further investigated. Western blot analysis showed elevated levels of the pro-apoptotic proteins Bax and Cleaved caspase-9 and reduced expression of the anti-apoptotic protein Bcl-2 in the LPS group compared with the Ctrl group (*p <* 0.05). In the FGF8± group, Bax and Cleaved-cas9 expression increased further, whereas Bcl-2 expression declined more markedly relative to the LPS group (Figure 3(A), (B), *p <* 0.05). Analysis of fibrosis-related markers demonstrated higher expression of α-SMA, TGF-β1 and COL1A1 in the LPS group than in the Ctrl group (*p <* 0.01), with further elevation observed in the FGF8± group (Figure 3(C), (D), *p <* 0.05). Masson trichrome staining revealed increased collagen deposition in LPS-treated lung tissue compared with the Ctrl group (*p <* 0.01), with more extensive collagen deposition detected in the FGF8± group (Figure 3(E), (F), *p <* 0.01). Overall, FGF8 haploinsufficiency intensified LPS-induced lung cell apoptosis and markedly enhanced early pulmonary fibrosis progression.

### 3.4. FGF8 overexpression alleviated LPS-induced ALI

**Figure 4.**
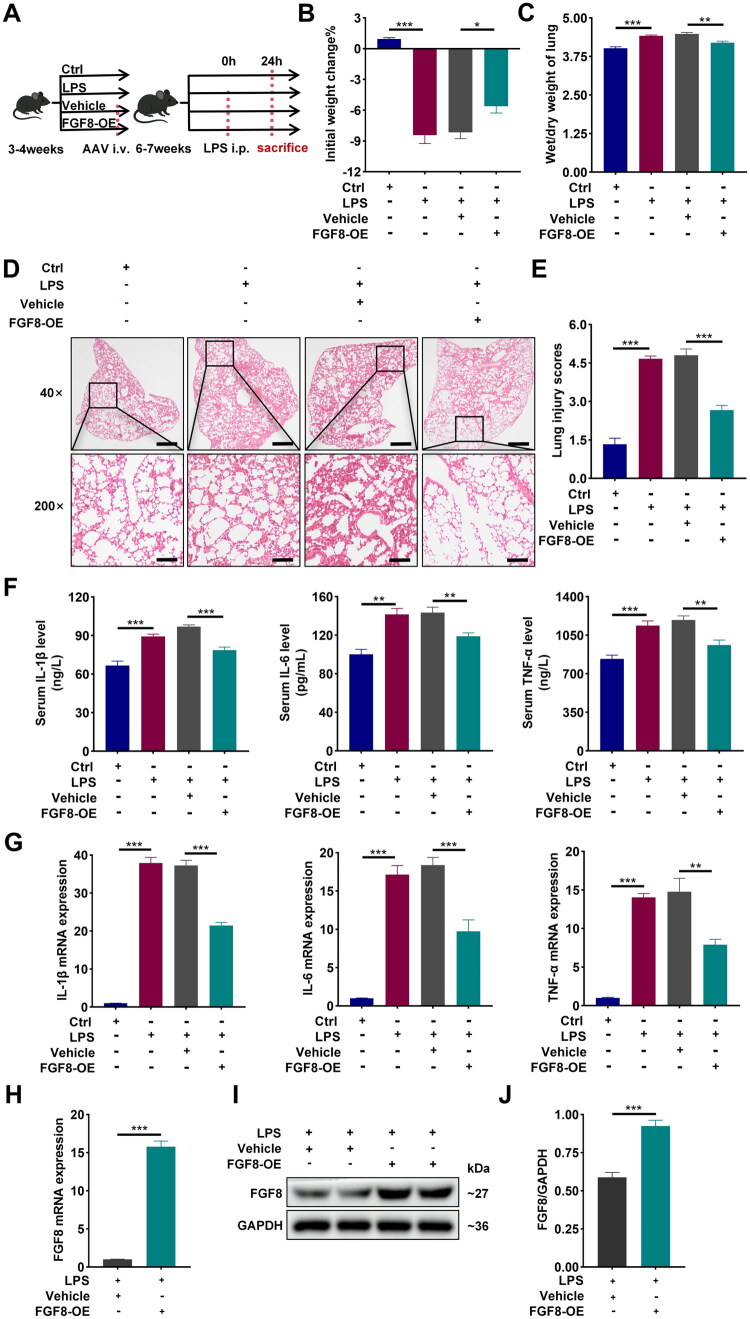
**FGF8 overexpression alleviated LPS-induced ALI**. **(A)** Animal experimental design diagram; **(B)** body weight change at 24 h following untreated/LPS intervention (*n* = 5); **(C)** lung wet-to-dry weight ratio (*n* = 5); **(D)** haematoxylin–eosin staining of paraffin-embedded lung sections (40×, scale bar = 500 μm; 200×, scale bar = 100 μm); **(E)** histopathological scoring of lung injury (*n* = 5); **(F)** ELISA-based assessment of serum IL-1β, IL-6 and TNF-α levels (*n* = 4–5); **(G)** qRT-PCR quantification of IL-1β, IL-6 and TNF-α mRNA expression in lung tissue (*n* = 4); **(H)** qRT-PCR assessment of FGF8 mRNA expression in lung tissue (*n* = 4–5); **(I, J)** Western blot analysis of FGF8 protein expression in lung tissue. Ctrl: control group; LPS: model group; Vehicle: negative vehicle group; FGF8-OE: FGF8 overexpression group. *p < 0.05, **p < 0.01, ***p < 0.001.

To determine the protective effect of FGF8 in ALI, an FGF8 overexpression strategy was applied in an LPS-induced mice model (Figure 4(A)). Pathological characteristics were comparable between the LPS and vehicle groups. Relative to the vehicle group, FGF8-OE mice exhibited attenuated weight loss (Figure 4(B), *p <* 0.05) and a reduced lung wet-to-dry weight ratio (Figure 4(C), *p <* 0.01), indicating partial mitigation of pulmonary oedema. H&E staining demonstrated that the vehicle group presented typical ALI-associated alterations, including alveolar collapse, thickened alveolar wall, infiltration of inflammatory cells and alveolar haemorrhage, whereas lung injury was markedly reduced in the FGF8-OE group (Figure 4(D)). Correspondingly, the pathological score declined significantly compared with the vehicle group (Figure 4(E), *p <* 0.001). In addition, serum levels of IL-1β, IL-6 and TNF-α (Figure 4(F), *p <* 0.01), as well as their mRNA expression in lung tissue, were significantly lower in the FGF8-OE group (Figure 4(G), *p <* 0.01). Successful overexpression of FGF8 was confirmed at both the mRNA and protein levels (Figure 4(H)–(J), *p <* 0.001). Collectively, these results indicate that FGF8 overexpression effectively ameliorated LPS-induced ALI.

### 3.5. FGF8 overexpression inhibited cell apoptosis and pulmonary fibrosis

**Figure 5.**
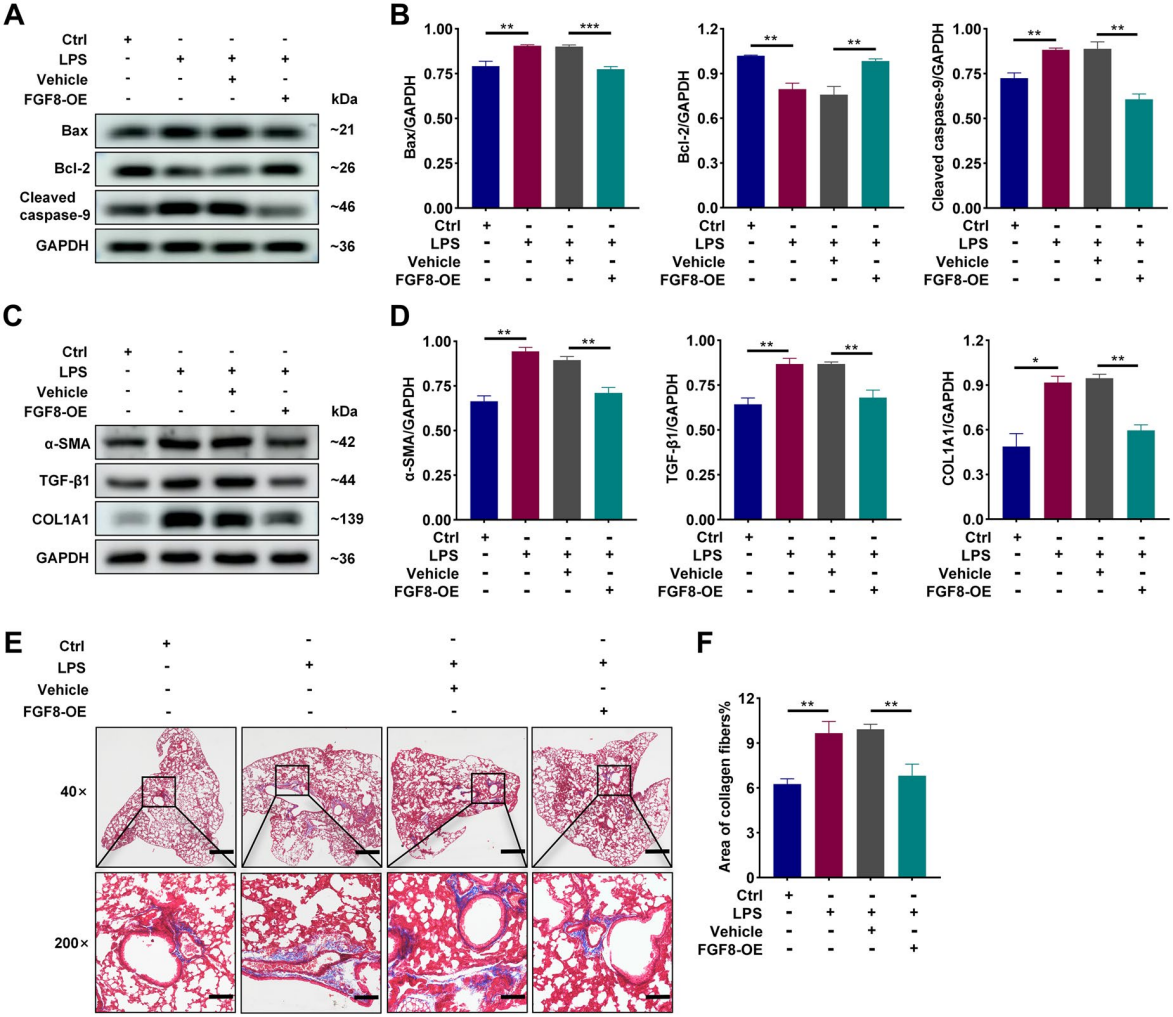
**FGF8 overexpression inhibited lung cell apoptosis and pulmonary fibrosis**. **(A, B)** Western blot analysis of Bax, Bcl2 and Cleaved caspase-9 protein levels in lung tissue; **(C, D)** Western blot evaluation of α-SMA, TGF-β1 and COL1A1 protein expression in lung tissue; **(E)** Masson staining of collagen fibers in paraffin-embedded lung sections (40×, scale bar = 500 μm; 200×, scale bar = 100 μm); **(F)** Semiquantitative analysis of collagen fiber deposition. *p < 0.05, **p < 0.01, ***p < 0.001.

The impact of FGF8 overexpression on apoptosis and early pulmonary fibrotic response in lung tissue was further assessed. Western blot analysis demonstrated that, relative to the vehicle group, the FGF8-OE group exhibited reduced expression of pro-apoptotic proteins Bax and Cleaved caspase-9 (*p <* 0.01), accompanied by elevated Bcl-2 expression (Figure 5(A), (B), *p <* 0.01). In addition, the levels of fibrosis-associated proteins a-SMA, TGF-β1 and COL1A1 were markedly diminished in the FGF8-OE group compared with the vehicle group (Figure 5(C), (D), *p <* 0.01). Masson staining further indicated substantially decreased collagen deposition in lung tissue following FGF8 overexpression (Figure 5(E), (F), *p <* 0.01). Collectively, the data indicate that FGF8 overexpression effectively suppresses LPS-induced lung cell apoptosis and early pulmonary fibrotic response.

### 3.6. FGF8 may have alleviated LPS-induced ALI *via* the PPARγ/ERK1/2 signalling pathway

**Figure 6.**
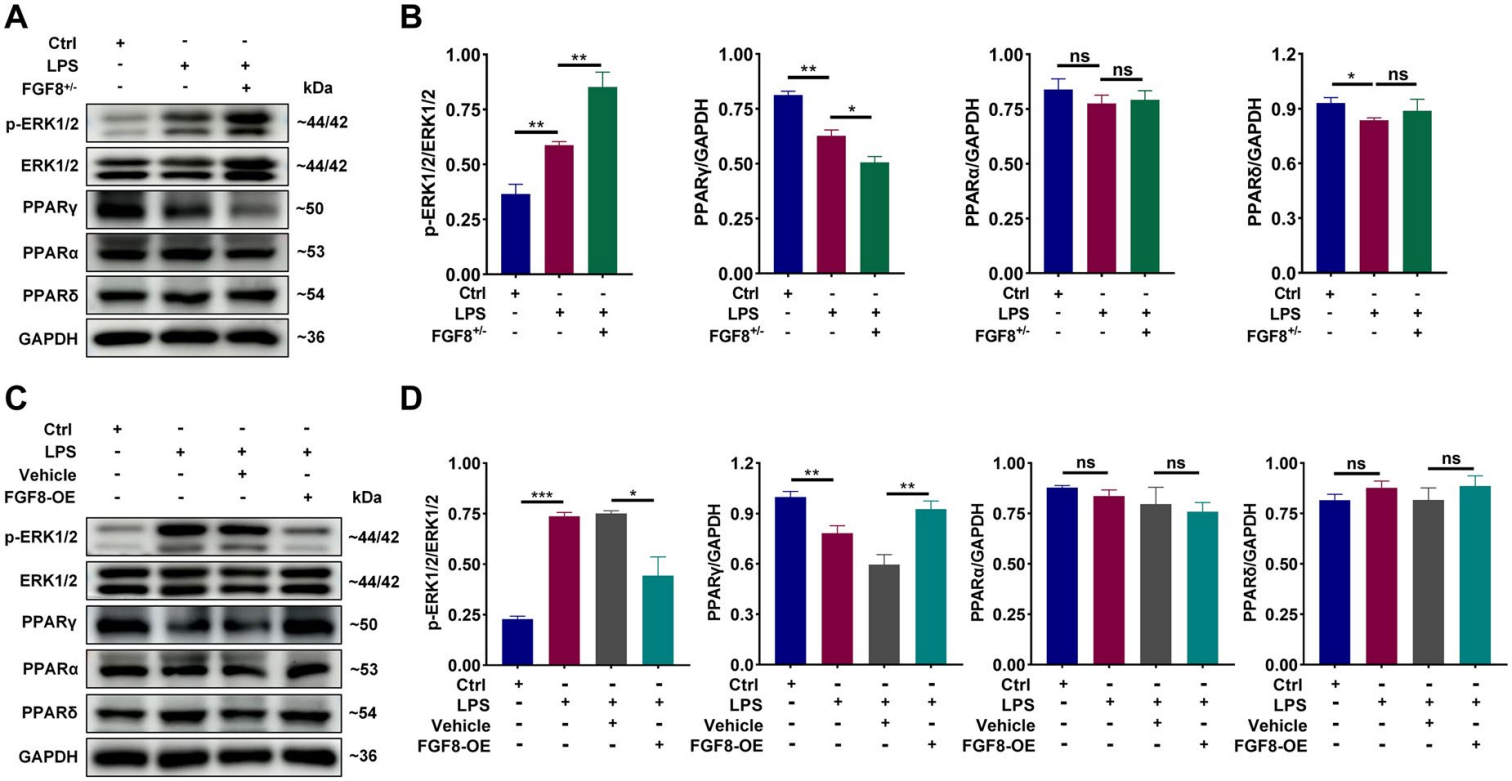
**FGF8 may have alleviated LPS-induced ALI** *via* **the PPARγ/ERK1/2 signalling pathway**. **(A–D)** Western blot analysis of PPARα, PPARδ, PPARγ, and phosphorylated ERK1/2 expression in lung tissue. ns: not statistically significant, *p < 0.05, **p < 0.01, ***p < 0.001.

Pathway enrichment analysis of RNA-seq data was performed to explore the potential protective mechanism of FGF8 against LPS-induced ALI. Relative to the Ctrl group, the LPS group exhibited 6,049 DEGs, including 2,204 upregulated and 3,845 downregulated transcripts. In comparison with the Vehicle group, the FGF8-OE group displayed 677 DEGs, with 285 upregulated and 392 downregulated (Fig. S1B). GO enrichment analysis revealed that these genes were predominantly associated with inflammatory response, immune system regulation, and bacterial response (Fig. S1C, S1D). KEGG pathway enrichment indicated a strong association between LPS-induced ALI and the MAPK signalling pathway, whereas FGF8 intervention appeared to modulate gene expression through the PPAR signalling pathway (Fig. S1E, 1 F). Western blotting further confirmed alterations in key regulatory molecules. In the FGF8 haploinsufficiency model, PPARγ expression in lung tissues was reduced in the LPS group compared with the Ctrl group (*p <* 0.01), while phosphorylated ERK1/2 levels were elevated (*p <* 0.01). In the FGF8± group, PPARγ expression showed an additional decrease relative to the LPS group (*p <* 0.05), accompanied by a further increase in phosphorylated ERK1/2 (*p <* 0.01), whereas PPARα and PPARδ levels remained unaltered (Figure 6(A), (B)). In contrast, within the FGF8 overexpression model, PPARγ was elevated (*p <* 0.01) and phosphorylated ERK1/2 was reduced (*p <* 0.05) in the FGF8-OE group compared with the Vehicle group, while PPARα and PPARδ expression remained stable (Figure 6(C), (D)). Collectively, these results indicate that the protective effect of FGF8 against LPS-induced ALI is accompanied by activation of PPARγ and inhibition of ERK1/2 signalling, suggesting a potential regulatory link between PPARγ and ERK1/2 that warrants further investigation.

### 3.7. PPARγ inhibitor GW9662 attenuated the alleviating effect of FGF8 on ALI

**Figure 7.**
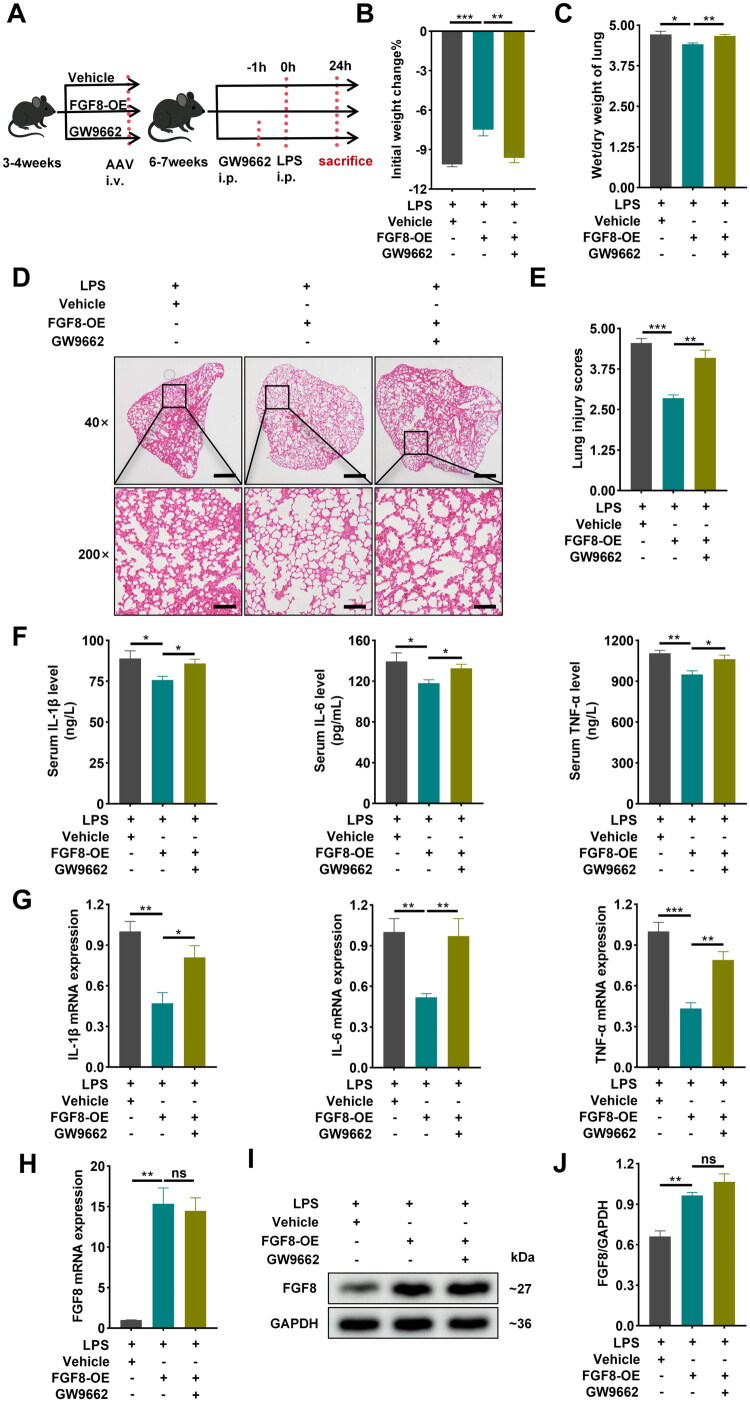
**PPARγ pathway inhibitor GW9662 attenuated the alleviating effect of FGF8 on ALI**. **(A)** Animal experimental design diagram; **(B)** body weight change at 24 h following untreated/LPS intervention (*n* = 5); **(C)** lung wet-to-dry weight ratio (*n* = 5); **(D)** haematoxylin–eosin staining of paraffin-embedded lung sections (40×, scale bar = 500 μm; 200×, scale bar = 100 μm); **(E)** histopathological scoring of lung injury (*n* = 5); **(F)** ELISA-based assessment of serum IL-1β, IL-6 and TNF-α levels (*n* = 4–5); **(G)** qRT-PCR quantification of IL-1β, IL-6 and TNF-α mRNA expression in lung tissue (*n* = 4); **(H)** qRT-PCR assessment of FGF8 mRNA expression in lung tissue (*n* = 4–5); **(I, J)** Western blot analysis of FGF8 protein expression in lung tissue. Vehicle: negative vehicle group; FGF8-OE: FGF8 overexpression group; GW9662: inhibitor group. *p < 0.05, **p < 0.01, ***p < 0.001.

We hypothesized that FGF8 exerts its protective effect by activating the PPARγ signalling pathway and inhibiting ERK1/2 signalling. To test this hypothesis, we treated FGF8‑overexpressing mice in an ALI model with the PPARγ‑specific antagonist GW9662 (Figure 7(A)). Compared with the FGF8-OE group, GW9662 treatment led to greater body weight loss (Figure 7(B), *p <* 0.01) and an increased lung wet-to-dry weight ratio (Figure 7(C), *p <* 0.01), indicating reversal of the FGF8-mediated reduction in pulmonary oedema. H&E analysis revealed aggravated histopathological damage, characterized by alveolar collapse, thickened alveolar walls, inflammatory infiltration and haemorrhage in the GW9662 group relative to FGF8-OE (Figure 7(D)), accompanied by a higher injury score (Figure 7(E), *p <* 0.01). Additionally, serum IL-1β, IL-6 and TNF-α concentrations were significantly elevated (Figure 7(F), *p <* 0.05), consistent with increased mRNA expression of these cytokines in lung tissue (Figure 7(G), *p <* 0.05). GW9662 administration did not alter FGF8 mRNA or protein expression (Figure 7(H)–(J)).

**Figure 8.**
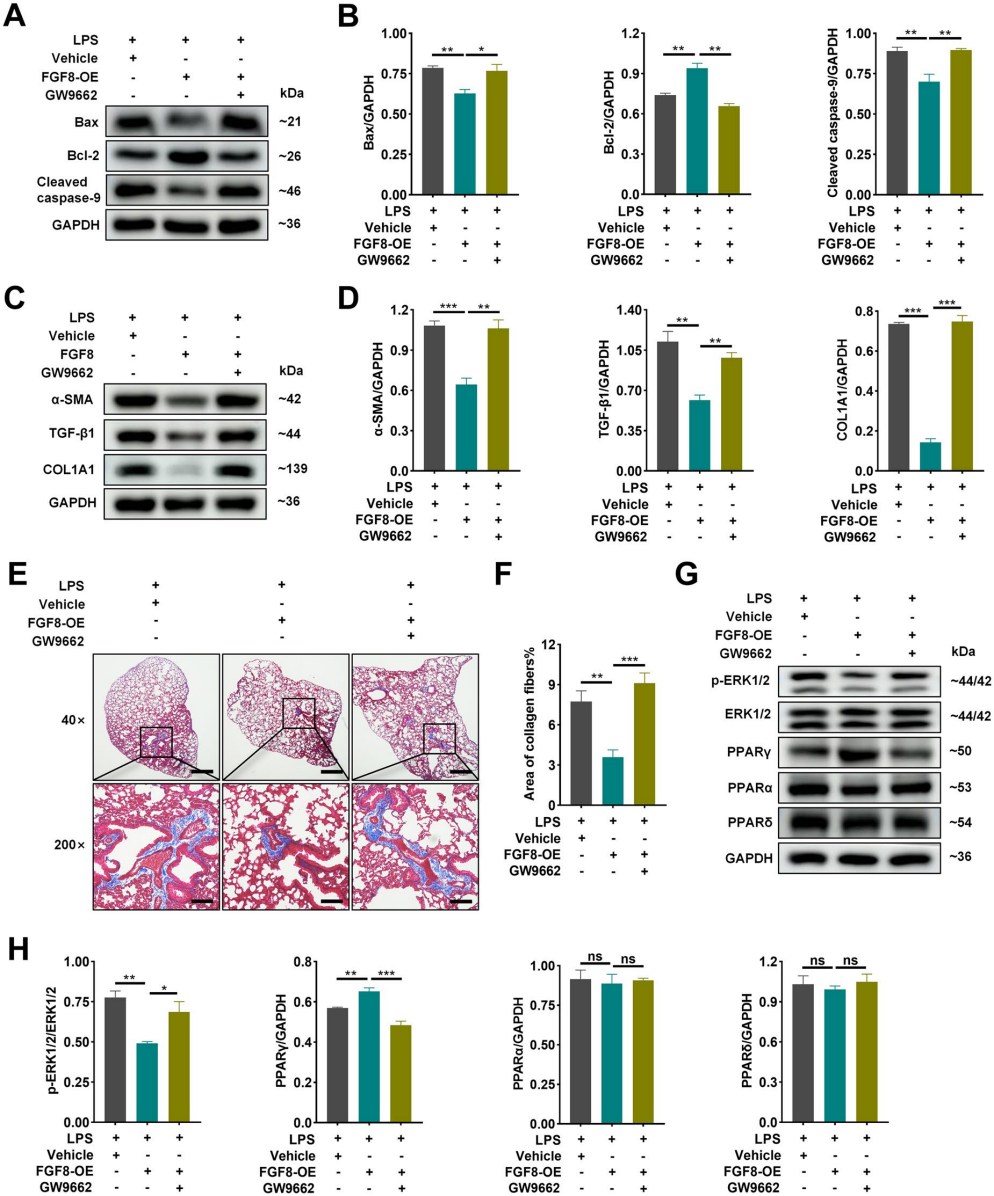
**PPARγ inhibitor GW9662 attenuated the alleviating effect of FGF8 on ALI. (A, B)** Western blot analysis of Bax, Bcl2 and Cleaved caspase-9 protein levels in lung tissue; **(C, D)** Western blot evaluation of α-SMA, TGF-β1 and COL1A1 protein expression in lung tissue; **(E)** Masson staining of collagen fibers in paraffin-embedded lung sections (40×, scale bar = 500 μm; 200×, scale bar = 100 μm); **(F)** semiquantitative analysis of collagen fiber deposition; **(G, H)** Western blot analysis of PPARα, PPARδ, PPARγ, and phosphorylated ERK1/2 levels in lung tissue. *p < 0.05, **p < 0.01, ***p < 0.001.

The Western blotting results demonstrated that, compared with FGF8-OE, GW9662 treatment upregulated Bax and Cleaved caspase-9 (*p <* 0.05) while reducing Bcl-2 expression (Figure 8(A), (B), *p <* 0.01), suggesting enhanced apoptosis. Fibrotic remodeling was also intensified, as indicated by elevated α-SMA, TGF-β1 and COL1A1 protein levels (Figure 8(C), (D), *p <* 0.01). Consistently, Masson staining showed a marked increase in collagen deposition in lung tissue following GW9662 treatment (Figure 8(E), (F), *p <* 0.001). Further Western blot analysis revealed that GW9662 significantly decreased PPARγ expression (*p <* 0.001) and increased ERK1/2 phosphorylation (*p <* 0.05), whereas PPARα and PPARδ levels were unaffected (Figure 8(G), (H)). Collectively, these results demonstrate that activation of PPARγ signalling is a key mechanism by which FGF8 exerts its lung-protective effects. Blockade of PPARγ by GW9662 diminished the inhibitory action of FGF8 on ERK1/2 signalling, thereby attenuating its alleviating effect on ALI.

## 4. Discussion

ALI/ARDS is a severe clinical condition marked by refractory hypoxemia and progressive respiratory failure [14], resulting in persistently high global morbidity and mortality rates [15]. Although advances have improved understanding of its pathophysiological mechanisms, the underlying pathogenesis remains poorly defined, and effective therapeutic strategies are still lacking worldwide [16]. Current research indicates that excessive inflammatory cascade activation, dysregulated apoptosis and progressive pulmonary fibrosis constitute the principal pathological mechanisms driving ALI development [17,18]. Increasing evidence indicates a protective function of FGF in ALI. FGF1 mitigates LPS-induced ALI by reducing inflammatory responses and oxidative stress [19]. rFGF4 attenuates tissue injury and suppresses apoptosis in lung tissue [20]. FGF18 alleviates hyperoxia-induced lung injury through inhibition of NF-κB signalling [21]. Deletion of FGF21 enhances lung inflammation following LPS exposure [22]. Nevertheless, the role of FGF8, another key member of the FGF family, in LPS-induced ALI has not been clarified. The present study demonstrates that FGF8 ameliorates LPS-induced lung tissue injury, excessive inflammation, aberrant apoptosis and early fibrotic changes by activating PPARγ and suppressing ERK1/2 signalling *in vivo*, thereby offering novel insights for potential therapeutic development in ALI.

Inflammation and apoptosis play pivotal roles in the pathogenesis of ALI. In this study, the levels of TNF-α, IL-1β and IL-6 were significantly elevated in both the serum and lung tissues of ALI mice, consistent with the previous reports [23,24]. Concurrently, FGF8 expression was upregulated in the lung, likely representing a protective compensatory response. In the FGF8 haploinsufficiency model, these pro‑inflammatory cytokines were further increased, whereas FGF8 overexpression markedly suppressed their levels, indicating that FGF8 effectively attenuates the inflammatory response. Regarding apoptosis, the pro‑apoptotic proteins Bax and Cleaved caspase‑9 were upregulated, while the anti‑apoptotic protein Bcl‑2 was downregulated in the lung tissues of ALI mice, reflecting enhanced apoptosis. FGF8 haploinsufficiency further elevated the ratio of pro‑apoptotic to anti‑apoptotic proteins, whereas FGF8 overexpression significantly reduced this ratio, demonstrating that FGF8 also mitigates apoptosis. Collectively, these results suggest that FGF8 alleviates LPS‑induced ALI by inhibiting both inflammatory and apoptotic pathways.

Acute pulmonary inflammation can trigger the proliferation and activation of lung fibroblasts, resulting in aberrant collagen deposition and accelerated pulmonary fibrosis [25]. In this study, the levels of α-SMA, TGF-β1 and COL1A1 were significantly elevated in the lung tissues of ALI mice, accompanied by increased collagen deposition. In the FGF8 haploinsufficiency model, these profibrotic factors were further upregulated, whereas FGF8 overexpression markedly reduced their levels, indicating that FGF8 attenuates pulmonary fibrosis. Notably, although pulmonary fibrosis is a chronic condition, the upregulation of α-SMA, TGF-β1 and COL1A1 together with collagen deposition observed in the ALI model reflects an early fibrotic response following acute lung injury [26,27]. The marked inhibitory effect of FGF8 on this early fibrotic response suggests that it may prevent the transition from acute injury to chronic fibrosis by blocking the initiation of the inflammatory–fibrotic cascade. Thus, the protective role of FGF8 extends beyond alleviating acute inflammation and apoptosis; it may also suppress subsequent irreversible pulmonary remodeling. This finding holds important clinical implications for preventing pulmonary fibrosis sequelae after ALI/ARDS.

PPARs, members of the nuclear receptor superfamily, comprise three isoforms: PPARα, PPARδ and PPARγ. They primarily regulate lipid and energy metabolism as well as inflammatory responses [28]. Among them, PPARγ exhibits anti-inflammatory and anti-fibrotic properties, particularly demonstrating therapeutic potential in pulmonary disorders [29]. Mitogen-activated protein kinases (MAPKs) govern essential cellular processes, including proliferation, differentiation, inflammatory responses and apoptosis. ERK, a central component of the MAPK family, acts as a common downstream effector of FGF signalling [30]. Recent evidence further indicates a regulatory function of MAPKs in fibrotic progression [31].

Mechanistically, substantial evidence supports the involvement of PPARγ and MAPK pathways, particularly ERK1/2, in ALI pathogenesis. Loss of PPARγ activity, such as through gene knockout, markedly diminishes its protective capacity, leading to intensified pulmonary inflammation [32] and increased collagen accumulation [33], whereas selective inhibition of MAPK signalling, including p38 MAPK, effectively mitigates LPS-induced lung injury [34]. Moreover, PPARγ and MAPK signalling are functionally interconnected rather than acting independently. PPARγ has been reported to modulate multiple MAPK components, including ERK, p38 and JNK [35]. Various bioactive agents with pulmonary protective effects, such as emodin, tenuigenin extract PRE and hydroxy safflower yellow A, exert therapeutic actions by enhancing PPARγ expression while suppressing MAPK phosphorylation, including that of p38, ERK and JNK [36–38]. FGF8, an essential signalling mediator, contributes to organogenesis, tissue repair, immune balance and regulation of cell fate through interactions with FGFRs [39,40]. However, its association with the PPARγ/ERK1/2 regulatory axis remains largely unexplored.

This study investigated the mechanistic association between FGF8 and the PPARγ/ERK1/2 signalling axis. FGF8 haploinsufficiency markedly reduced PPARγ expression while enhancing ERK1/2 phosphorylation, indicating weakened suppression of ERK1/2 activity and suggesting enhanced tissue injury. In contrast, FGF8 overexpression produced the inverse response, characterized by elevated PPARγ expression and reduced ERK1/2 phosphorylation. These results indicate that FGF8 may negatively modulate ERK1/2 signalling through positive regulation of PPARγ. To clarify the involvement of PPARγ in this regulatory cascade, the PPARγ antagonist GW9662 was employed. In the setting of FGF8 overexpression, pharmacological inhibition of PPARγ resulted in a marked restoration of ERK1/2 phosphorylation, which had been diminished by FGF8 overexpression. Correspondingly, lung injury, inflammatory response, apoptosis and early fibrotic response in the GW9662 group were markedly more severe than in the FGF8-OE group, indicating that PPARγ inhibition diminished the protective effects of FGF8. Notably, although GW9662 partially reversed the protective role of FGF8, the extent of injury remained less severe than in the vehicle group. This outcome implies that FGF8 may additionally engage alternative signalling pathways beyond the PPARγ/ERK1/2 axis in mitigating ALI.

Despite the innovative insights offered in this study, certain limitations warrant consideration. Firstly, mechanistic studies of FGF8 are limited to mouse models. Future *in vitro* experiments are needed to directly examine the effect of FGF8 on PPARγ activity in various cell types (such as macrophages, epithelial cells and fibroblasts) to elucidate the cell‑type specificity of its mechanism. Secondly, the limited RNA-seq sample size may have obscured critical alterations in signalling pathways due to inter-individual variability, indicating that a larger cohort is necessary to improve the statistical robustness of DEGs. Finally, although studies have demonstrated that FGF8 attenuates LPS-induced ALI by activating PPARγ and inhibiting ERK1/2 phosphorylation, the interplay among these three factors has not been fully clarified.

## 5. Conclusions

In conclusion, this study demonstrates that FGF8 alleviates LPS‑induced ALI by activating PPARγ signalling and inhibiting the ERK1/2 pathway, thereby offering a novel molecular intervention strategy for ALI/ARDS treatment. The key significance of this work lies in demonstrating that FGF8 exerts anti‑inflammatory, anti‑apoptotic and early anti‑fibrotic effects, underscoring the therapeutic potential of developmentally related molecules in tissue injury repair. Moreover, given that the PPARγ and ERK1/2 pathways are also implicated in the pathogenesis of other pulmonary diseases such as asthma and pulmonary fibrosis, these findings may provide new insights for the development of therapeutic targets for these conditions.

## Supplementary Material

Supplementary figure 1.tif

## Data Availability

All data and materials are available from the corresponding author on reasonable request.

## ARRIVE guidelines

All authors have adhered to ARRIVE guidelines.
